# (2*S*,3*R*)-3-(2-Bromo­phen­yl)-2-nitro-2,3,6,7-tetra­hydro-1-benzo­furan-4(5*H*)-one

**DOI:** 10.1107/S1600536813017698

**Published:** 2013-07-03

**Authors:** Yifeng Wang, Liuliu Lou, Kun Dong, Danqian Xu

**Affiliations:** a Catalytic Hydrogenation Research Center, Zhejiang University of Technology, Hangzhou 310014, People’s Republic of China

## Abstract

The title compound, C_14_H_12_BrNO_4_, has two chiral C atoms. The C atom next to the O atom in the di­hydro­furan ring has an *S* configuration, while the adjacent chiral C atom has an *R* configuration. The cyclo­hex-2-enone and di­hydro­furan rings both adopt envelope conformations, with the flap atoms (middle CH_2_ in cyclo­hex-2-enone and NO_2_-substituted C in di­hydro­furan) lying 0.612 (3) and 0.295 (2) Å, respectively, from the mean plane of the remaining atoms. The dihedral angle between the mean planes of the furan and benzene rings is 80.0 (3)°. In the crystal, the mol­ecules are linked by C—H⋯O inter­actions, generating a three-dimensional network.

## Related literature

For global background on functionalized 2,3-di­hydro­furans, see: Fan *et al.* (2010 ▶); Rueping *et al.* (2010 ▶). The absolute configuration was assigned by the method of Flack (1983 ▶).
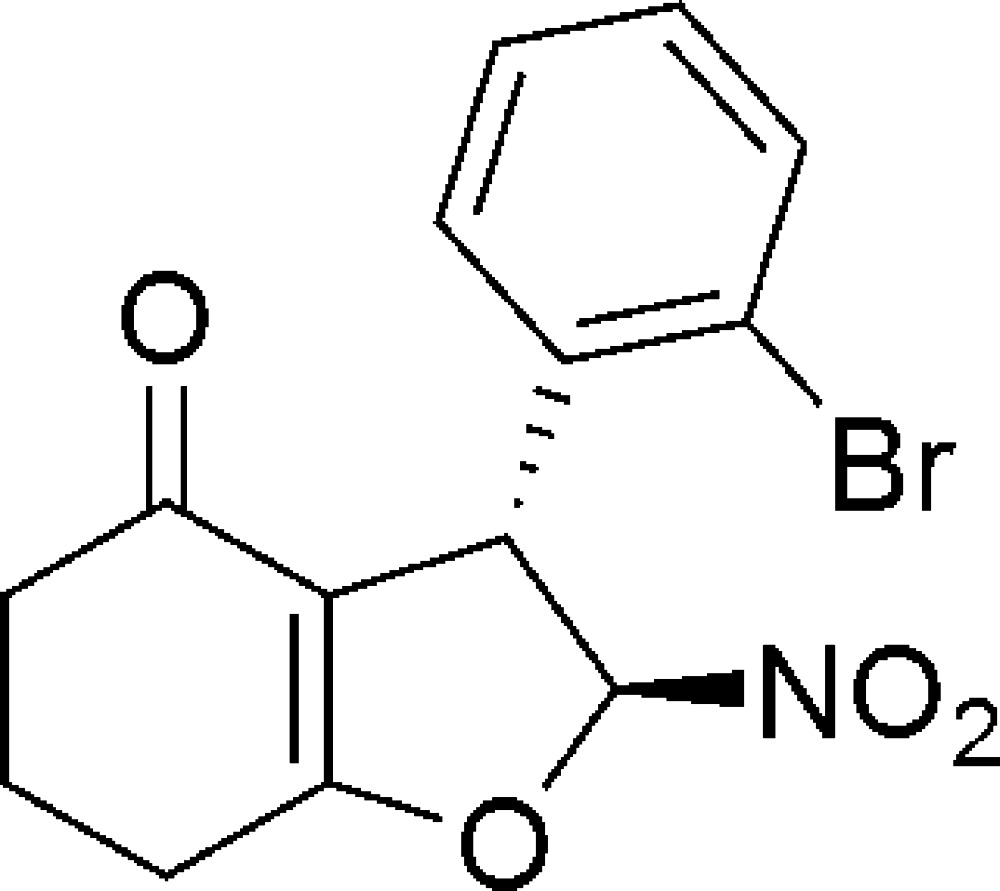


## Experimental

### 

#### Crystal data

C_14_H_12_BrNO_4_
*M* *_r_* = 338.16
Orthorhombic, 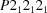

*a* = 7.2162 (8) Å
*b* = 7.3372 (8) Å
*c* = 25.9727 (13) Å
*V* = 1375.2 (2) Å^3^
*Z* = 4
Mo *K*α radiation
μ = 3.00 mm^−1^
*T* = 296 K
0.54 × 0.31 × 0.23 mm

#### Data collection

Rigaku R-AXIS RAPID/ZJUG diffractometer
Absorption correction: multi-scan (*ABSCOR*; Higashi, 1995 ▶) *T*_min_ = 0.334, *T*_max_ = 0.505
10897 measured reflections
2548 independent reflections
1456 reflections with *I* > 2σ(*I*)
*R*_int_ = 0.068

#### Refinement

*R*[*F* ^2^ > 2σ(*F* ^2^)] = 0.059
*wR*(*F* ^2^) = 0.125
*S* = 1.00
2548 reflections
182 parameters
H-atom parameters constrained
Δρ_max_ = 0.51 e Å^−3^
Δρ_min_ = −0.57 e Å^−3^
Absolute structure: Flack (1983 ▶), 1041 Friedel pairs
Flack parameter: 0.03 (2)

### 

Data collection: *PROCESS-AUTO* (Rigaku, 2006 ▶); cell refinement: *PROCESS-AUTO*; data reduction: *CrystalStructure* (Rigaku,2007 ▶); program(s) used to solve structure: *SHELXS97* (Sheldrick, 2008 ▶); program(s) used to refine structure: *SHELXL97* (Sheldrick, 2008 ▶); molecular graphics: *ORTEP-3 for Windows* (Farrugia, 2012 ▶); software used to prepare material for publication: *WinGX* (Farrugia, 2012 ▶).

## Supplementary Material

Crystal structure: contains datablock(s) global, I. DOI: 10.1107/S1600536813017698/pk2489sup1.cif

Structure factors: contains datablock(s) I. DOI: 10.1107/S1600536813017698/pk2489Isup2.hkl

Supplementary material file. DOI: 10.1107/S1600536813017698/pk2489Isup3.cml

Additional supplementary materials:  crystallographic information; 3D view; checkCIF report

## Figures and Tables

**Table 1:** Hydrogen-bond geometry (Å, °)

*D*—H⋯*A*	*D*—H	H⋯*A*	*D*⋯*A*	*D*—H⋯*A*
C1—H1⋯O1^i^	0.98	2.51	3.486 (12)	171
C7—H7*A*⋯O3^ii^	0.97	2.66	3.403 (12)	134
C7—H7*B*⋯O3^iii^	0.97	2.52	3.480 (13)	172

Symmetry codes: (i) 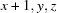; (ii) 
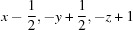
; (iii) 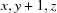.

**Fig. 1.**
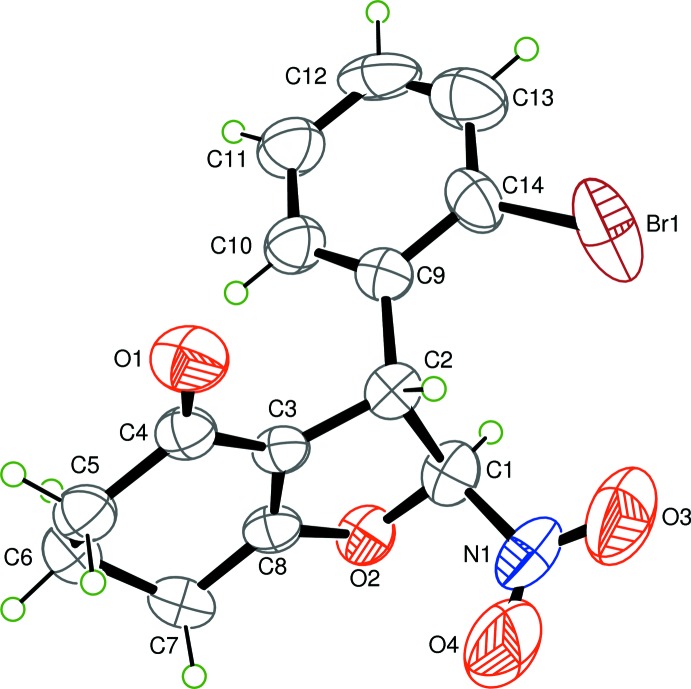
The asymmetric unit of the structure of the title compound. Displacement ellipsoids are drawn at the 50% probability level.

**Fig. 2.**
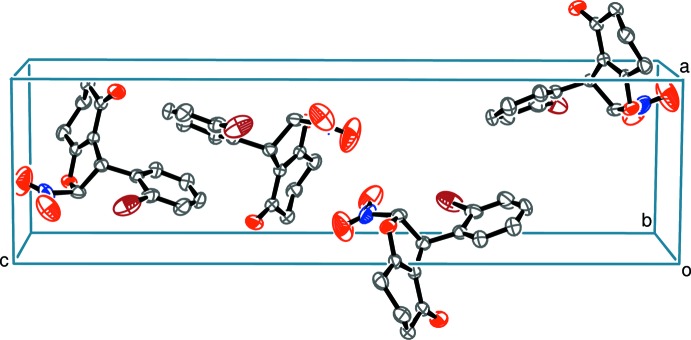
The molecular packing of the title compounds.

## supplementary crystallographic information

### Comment

The highly functionalized 2,3-dihydrofurans are very important compounds that may serve as precursors for the construction of pharmacologically important chemicals. Organocatalytic asymmetric reactions have been used as efficient tools for the synthesis of chiral compounds under mild conditions. The title compound, which was readily synthesized by the organocatalytic Michael-S_N_2 reaction of cyclohexane-1,3-dione to (*E*)-1-bromo-2-(2-bromo-2-nitrovinyl)benzene, could act as an intermediate in organic and natural product synthesis. In this article, the crystal structure of the title compound (2*S*,3*R*)-3-(2-bromophenyl)-2-nitro-2,3,6,7- tetrahydrobenzofuran-4(5*H*)-one is described (Fig. 1). The structure has two chiral centers. The carbon next to the oxygen atom in the dihydrofuran ring has *S* configuration, while the adjacent chiral carbon atom has *R* configuration. Both the cyclohex-2-enone ring and dihydrofurane ring adopt envelope conformations, with the flap carbon atom lying 0.612 (3) Å and 0.295 (2) Å respectively on either side of the mean plane of the remaining fused-ring atoms. The dihedral angle between the mean plane of the furan ring and the benzene ring is 80.0 (3)°.

### Experimental

To a solution of cyclohexane-1,3-dione (1.2 mmol) and (*E*)-1-bromo-2-(2-bromo-2-nitrovinyl)benzene (1 mmol) in CHCl_3_ (3 ml) was added (0.025 mmol) 1-(3,5-bis(trifluoromethyl)phenyl)-3-((*S*) -(6-methoxyquinolin-4-yl)((2*S*,4*S*,8*R*)-8-vinylquinuclidin-2 -yl)methyl)thiourea as catalyst and DIPEA (0.3 mmol) as the base. The mixture was stirred at room temperature for 12 h (monitored by TLC). Then the solvent was evaporated under vacuum, and the residue was purified by flash column chromatography (silica gel, Hex/AcOEt, *v*/*v*, 3:1) giving the title compound. Single crystals were obtained by slow evaporation of a CH_2_Cl_2_ and *i*PrOH solution (*v*/*v*, 1:1).

### Refinement

H atoms were placed in calculated positions with C—H = 0.98 Å (R_3_CH), C—H = 0.97 Å (R_2_CH_2_), C—H = 0.93 Å (aromatic). All H atoms included in the final cycles of refinement using a riding model, with *U*_iso_(H) = 1.2*U*_eq_ of the carrier atoms.

### Crystal data

**Table:**

C_14_H_12_BrNO_4_	*F*(000) = 680
*M**_r_* = 338.16	*D*_x_ = 1.633 Mg m^−^^3^
Orthorhombic, *P*2_1_2_1_2_1_	Mo *K*α radiation, λ = 0.71073 Å
Hall symbol: P 2ac 2ab	Cell parameters from 6447 reflections
*a* = 7.2162 (8) Å	θ = 3.1–27.4°
*b* = 7.3372 (8) Å	µ = 3.00 mm^−^^1^
*c* = 25.9727 (13) Å	*T* = 296 K
*V* = 1375.2 (2) Å^3^	Needle, colorless
*Z* = 4	0.54 × 0.31 × 0.23 mm

### Data collection

**Table:**

Rigaku R-AXIS RAPID/ZJUG diffractometer	2548 independent reflections
Radiation source: rotating anode	1456 reflections with *I* > 2σ(*I*)
Graphite monochromator	*R*_int_ = 0.068
Detector resolution: 10.00 pixels mm^-1^	θ_max_ = 25.5°, θ_min_ = 3.1°
ω scans	*h* = −8→8
Absorption correction: multi-scan (*ABSCOR*; Higashi, 1995)	*k* = −8→7
*T*_min_ = 0.334, *T*_max_ = 0.505	*l* = −31→31
10897 measured reflections	

### Refinement

**Table:**

Refinement on *F*^2^	Hydrogen site location: inferred from neighbouring sites
Least-squares matrix: full	H-atom parameters constrained
*R*[*F*^2^ > 2σ(*F*^2^)] = 0.059	*w* = 1/[σ^2^(*F*_o_^2^) + (0.0081*P*)^2^ + 2.5658*P*] where *P* = (*F*_o_^2^ + 2*F*_c_^2^)/3
*wR*(*F*^2^) = 0.125	(Δ/σ)_max_ < 0.001
*S* = 1.00	Δρ_max_ = 0.51 e Å^−^^3^
2548 reflections	Δρ_min_ = −0.57 e Å^−^^3^
182 parameters	Extinction correction: *SHELXL97* (Sheldrick, 2008), Fc^*^=kFc[1+0.001xFc^2^λ^3^/sin(2θ)]^-1/4^
0 restraints	Extinction coefficient: 0.0117 (17)
Primary atom site location: structure-invariant direct methods	Absolute structure: Flack (1983), 1041 Friedel pairs
Secondary atom site location: difference Fourier map	Flack parameter: 0.03 (2)

### Special details

**Table:**

Geometry. All e.s.d.'s (except the e.s.d. in the dihedral angle between two l.s. planes) are estimated using the full covariance matrix. The cell e.s.d.'s are taken into account individually in the estimation of e.s.d.'s in distances, angles and torsion angles; correlations between e.s.d.'s in cell parameters are only used when they are defined by crystal symmetry. An approximate (isotropic) treatment of cell e.s.d.'s is used for estimating e.s.d.'s involving l.s. planes.
Refinement. Refinement of *F*^2^ against ALL reflections. The weighted *R*-factor *wR* and goodness of fit *S* are based on *F*^2^, conventional *R*-factors *R* are based on *F*, with *F* set to zero for negative *F*^2^. The threshold expression of *F*^2^ > σ(*F*^2^) is used only for calculating *R*-factors(gt) *etc*. and is not relevant to the choice of reflections for refinement. *R*-factors based on *F*^2^ are statistically about twice as large as those based on *F*, and *R*- factors based on ALL data will be even larger.

### Fractional atomic coordinates and isotropic or equivalent isotropic displacement parameters (Å^2^)

**Table:**

	*x*	*y*	*z*	*U*_iso_*/*U*_eq_	
C1	0.7398 (10)	0.3494 (8)	0.5784 (2)	0.0619 (16)	
H1	0.8610	0.3377	0.5952	0.074*	
C2	0.5793 (7)	0.3053 (8)	0.6169 (2)	0.0516 (15)	
H2	0.5270	0.1847	0.6098	0.062*	
C3	0.4449 (7)	0.4531 (9)	0.6016 (2)	0.0492 (14)	
C4	0.2478 (9)	0.4690 (8)	0.6141 (2)	0.0569 (14)	
C5	0.1536 (9)	0.6322 (9)	0.5902 (2)	0.0660 (19)	
H5A	0.0550	0.6715	0.6131	0.079*	
H5B	0.0968	0.5944	0.5581	0.079*	
C6	0.2792 (9)	0.7952 (8)	0.5794 (2)	0.0677 (18)	
H6A	0.3187	0.8488	0.6117	0.081*	
H6B	0.2102	0.8867	0.5604	0.081*	
C7	0.4499 (9)	0.7389 (9)	0.5482 (2)	0.0660 (18)	
H7A	0.4154	0.7167	0.5126	0.079*	
H7B	0.5416	0.8355	0.5489	0.079*	
C8	0.5265 (8)	0.5737 (9)	0.5708 (2)	0.0506 (15)	
C9	0.6438 (8)	0.3183 (8)	0.6723 (2)	0.0532 (15)	
C10	0.6320 (9)	0.4785 (10)	0.7000 (3)	0.0721 (19)	
H10	0.5734	0.5781	0.6850	0.087*	
C11	0.7034 (10)	0.4972 (12)	0.7491 (3)	0.085 (2)	
H11	0.6976	0.6076	0.7666	0.102*	
C12	0.7845 (11)	0.3438 (16)	0.7712 (3)	0.098 (3)	
H12	0.8302	0.3514	0.8046	0.118*	
C13	0.7987 (11)	0.1840 (14)	0.7455 (4)	0.096 (3)	
H13	0.8524	0.0836	0.7615	0.116*	
C14	0.7336 (10)	0.1696 (8)	0.6957 (3)	0.0698 (18)	
N1	0.7257 (12)	0.2243 (10)	0.5319 (3)	0.091 (2)	
O1	0.1679 (6)	0.3594 (7)	0.64055 (19)	0.0816 (14)	
O2	0.7098 (5)	0.5307 (6)	0.56089 (15)	0.0623 (12)	
O3	0.7909 (12)	0.0725 (9)	0.5383 (3)	0.147 (3)	
O4	0.6648 (14)	0.2703 (15)	0.4940 (3)	0.211 (5)	
Br1	0.75326 (16)	−0.05596 (11)	0.66299 (5)	0.1322 (6)	

### Atomic displacement parameters (Å^2^)

**Table:**

	*U*^11^	*U*^22^	*U*^33^	*U*^12^	*U*^13^	*U*^23^
C1	0.042 (3)	0.072 (4)	0.073 (4)	−0.007 (4)	0.004 (4)	−0.006 (3)
C2	0.046 (3)	0.053 (3)	0.057 (4)	−0.010 (3)	0.002 (3)	−0.004 (3)
C3	0.046 (3)	0.064 (4)	0.037 (3)	−0.008 (3)	−0.001 (3)	−0.002 (3)
C4	0.051 (3)	0.069 (4)	0.051 (3)	−0.004 (4)	−0.005 (3)	−0.003 (3)
C5	0.057 (4)	0.090 (5)	0.051 (4)	0.003 (4)	−0.007 (3)	0.000 (4)
C6	0.067 (5)	0.062 (4)	0.074 (4)	0.004 (4)	−0.013 (4)	0.005 (3)
C7	0.072 (5)	0.068 (4)	0.058 (4)	−0.008 (4)	−0.014 (4)	0.012 (3)
C8	0.050 (3)	0.061 (4)	0.040 (3)	−0.010 (3)	−0.005 (3)	−0.003 (3)
C9	0.045 (3)	0.059 (4)	0.056 (4)	−0.006 (3)	0.001 (3)	0.006 (3)
C10	0.070 (4)	0.087 (5)	0.059 (4)	0.006 (4)	−0.003 (4)	−0.006 (4)
C11	0.082 (5)	0.112 (7)	0.061 (5)	0.004 (5)	−0.009 (4)	−0.012 (4)
C12	0.070 (6)	0.180 (9)	0.044 (4)	−0.010 (6)	−0.014 (4)	0.026 (6)
C13	0.071 (6)	0.123 (7)	0.095 (7)	0.000 (6)	−0.016 (5)	0.036 (6)
C14	0.050 (4)	0.074 (4)	0.085 (5)	0.009 (4)	−0.005 (4)	0.014 (4)
N1	0.098 (5)	0.091 (5)	0.082 (5)	0.002 (5)	0.033 (4)	−0.027 (4)
O1	0.056 (3)	0.104 (4)	0.085 (3)	−0.014 (3)	0.010 (2)	0.022 (3)
O2	0.047 (3)	0.071 (3)	0.068 (3)	−0.009 (2)	0.012 (2)	0.001 (2)
O3	0.195 (8)	0.093 (4)	0.152 (6)	−0.001 (6)	0.066 (6)	−0.038 (4)
O4	0.293 (12)	0.231 (9)	0.108 (6)	0.139 (9)	−0.073 (7)	−0.096 (6)
Br1	0.1192 (8)	0.0727 (5)	0.2046 (12)	0.0255 (6)	−0.0517 (9)	0.0010 (6)

### Geometric parameters (Å, º)

**Table:**

C1—O2	1.423 (7)	C7—C8	1.455 (8)
C1—N1	1.520 (8)	C7—H7A	0.9700
C1—C2	1.563 (8)	C7—H7B	0.9700
C1—H1	0.9800	C8—O2	1.384 (7)
C2—C3	1.507 (8)	C9—C10	1.381 (9)
C2—C9	1.517 (8)	C9—C14	1.407 (8)
C2—H2	0.9800	C10—C11	1.382 (9)
C3—C8	1.332 (8)	C10—H10	0.9300
C3—C4	1.463 (8)	C11—C12	1.393 (11)
C4—O1	1.205 (6)	C11—H11	0.9300
C4—C5	1.510 (8)	C12—C13	1.352 (11)
C5—C6	1.527 (8)	C12—H12	0.9300
C5—H5A	0.9700	C13—C14	1.380 (10)
C5—H5B	0.9700	C13—H13	0.9300
C6—C7	1.531 (9)	C14—Br1	1.866 (7)
C6—H6A	0.9700	N1—O4	1.130 (9)
C6—H6B	0.9700	N1—O3	1.221 (9)
			
O2—C1—N1	107.4 (5)	C8—C7—H7A	110.0
O2—C1—C2	106.5 (5)	C6—C7—H7A	110.0
N1—C1—C2	109.5 (5)	C8—C7—H7B	110.0
O2—C1—H1	111.1	C6—C7—H7B	110.0
N1—C1—H1	111.1	H7A—C7—H7B	108.4
C2—C1—H1	111.1	C3—C8—O2	112.5 (6)
C3—C2—C9	113.7 (5)	C3—C8—C7	129.0 (6)
C3—C2—C1	99.3 (5)	O2—C8—C7	118.5 (5)
C9—C2—C1	111.5 (5)	C10—C9—C14	117.6 (6)
C3—C2—H2	110.7	C10—C9—C2	121.9 (6)
C9—C2—H2	110.7	C14—C9—C2	120.2 (6)
C1—C2—H2	110.7	C9—C10—C11	122.8 (7)
C8—C3—C4	120.6 (6)	C9—C10—H10	118.6
C8—C3—C2	110.6 (5)	C11—C10—H10	118.6
C4—C3—C2	128.7 (5)	C10—C11—C12	117.2 (7)
O1—C4—C3	122.5 (6)	C10—C11—H11	121.4
O1—C4—C5	123.2 (6)	C12—C11—H11	121.4
C3—C4—C5	114.3 (6)	C13—C12—C11	121.9 (7)
C4—C5—C6	115.4 (5)	C13—C12—H12	119.0
C4—C5—H5A	108.4	C11—C12—H12	119.0
C6—C5—H5A	108.4	C12—C13—C14	120.2 (8)
C4—C5—H5B	108.4	C12—C13—H13	119.9
C6—C5—H5B	108.4	C14—C13—H13	119.9
H5A—C5—H5B	107.5	C13—C14—C9	120.1 (7)
C5—C6—C7	111.4 (5)	C13—C14—Br1	118.0 (6)
C5—C6—H6A	109.4	C9—C14—Br1	121.7 (5)
C7—C6—H6A	109.4	O4—N1—O3	122.7 (9)
C5—C6—H6B	109.4	O4—N1—C1	122.6 (8)
C7—C6—H6B	109.4	O3—N1—C1	114.7 (8)
H6A—C6—H6B	108.0	C8—O2—C1	107.4 (5)
C8—C7—C6	108.5 (5)		
			
O2—C1—C2—C3	17.9 (6)	C1—C2—C9—C10	90.7 (7)
N1—C1—C2—C3	−98.0 (6)	C3—C2—C9—C14	165.2 (5)
O2—C1—C2—C9	−102.2 (6)	C1—C2—C9—C14	−83.6 (7)
N1—C1—C2—C9	142.0 (6)	C14—C9—C10—C11	−0.2 (10)
C9—C2—C3—C8	107.2 (6)	C2—C9—C10—C11	−174.6 (6)
C1—C2—C3—C8	−11.3 (6)	C9—C10—C11—C12	−2.2 (11)
C9—C2—C3—C4	−76.5 (7)	C10—C11—C12—C13	2.0 (13)
C1—C2—C3—C4	165.1 (5)	C11—C12—C13—C14	0.7 (14)
C8—C3—C4—O1	177.6 (6)	C12—C13—C14—C9	−3.2 (12)
C2—C3—C4—O1	1.6 (9)	C12—C13—C14—Br1	−178.8 (7)
C8—C3—C4—C5	−1.4 (8)	C10—C9—C14—C13	3.0 (10)
C2—C3—C4—C5	−177.5 (5)	C2—C9—C14—C13	177.5 (6)
O1—C4—C5—C6	153.7 (6)	C10—C9—C14—Br1	178.4 (5)
C3—C4—C5—C6	−27.3 (7)	C2—C9—C14—Br1	−7.1 (8)
C4—C5—C6—C7	52.2 (7)	O2—C1—N1—O4	−14.4 (12)
C5—C6—C7—C8	−45.7 (7)	C2—C1—N1—O4	101.0 (11)
C4—C3—C8—O2	−176.3 (5)	O2—C1—N1—O3	163.3 (7)
C2—C3—C8—O2	0.4 (7)	C2—C1—N1—O3	−81.4 (9)
C4—C3—C8—C7	4.9 (10)	C3—C8—O2—C1	12.2 (6)
C2—C3—C8—C7	−178.4 (6)	C7—C8—O2—C1	−168.9 (5)
C6—C7—C8—C3	19.8 (9)	N1—C1—O2—C8	98.3 (6)
C6—C7—C8—O2	−158.9 (5)	C2—C1—O2—C8	−19.0 (6)
C3—C2—C9—C10	−20.5 (8)		

### Hydrogen-bond geometry (Å, º)

**Table:**

*D*—H···*A*	*D*—H	H···*A*	*D*···*A*	*D*—H···*A*
C1—H1···O1^i^	0.98	2.51	3.486 (12)	171
C7—H7*A*···O3^ii^	0.97	2.66	3.403 (12)	134
C7—H7*B*···O3^iii^	0.97	2.52	3.480 (13)	172

Symmetry codes: (i) *x*+1, *y*, *z*; (ii) *x*−1/2, −*y*+1/2, −*z*+1; (iii) *x*, *y*+1, *z*.
